# Characteristics of metabolites analysis for patients with granulomatous lobular mastitis

**DOI:** 10.3389/fcimb.2025.1514315

**Published:** 2025-06-10

**Authors:** Qiuying Dai, Yue Zhou, Jingjing Wu, Mingjuan Liao

**Affiliations:** ^1^ Department of Traditional Chinese Medicine, The Ninth People’s Hospital Affiliated to Shanghai Jiaotong University School of Medicine, Shanghai, China; ^2^ Department of Breast Surgery, LongHua Hospital Shanghai University of Traditional Chinese Medicine, Shanghai, China

**Keywords:** granulomatous lobular mastitis, short-chain fatty acid, gut microbiota, non-puerperal, metabolite

## Abstract

**Background:**

Granulomatous lobular mastitis (GLM) is a type of non-puerperal mastitis (NPM) associated with autoimmune factors. Previous studies suggest that gut microbiota dysbiosis may play a role in the pathological process of GLM; however, the specific role of gut microbiota metabolites in this process remains unclear. Therefore, this study aimed to investigate the gut metabolic characteristics of short-chain fatty acid (SCFA) in patients with GLM, a type of NPM.

**Methods:**

Stool samples were collected from 35 patients with GLM and 26 healthy control (HC) subjects. These samples underwent targeted metabolomic analysis to measure SCFA and 16s rRNA high-throughput sequencing to assess gut microbiota composition and differences between the groups.

**Results:**

Gas chromatography-mass spectrometry(GC-MS) analysis revealed that the content of SCFA-butanoic acid in the feces of patients with GLM was higher than that in the HC group. Notably, significant differences in metabolic pathways were observed between the HC and GLM groups. High-throughput sequencing results showed that the richness and diversity of gut microbiota in patients with GLM were significantly lower than those in healthy individuals. In addition, 53 bacterial species were found to differ significantly in abundance between the two groups. Moreover, the level of isohexanoic acid in the feces of patients with GLM with recurrence disease was significantly higher than that of patients without recurrence.

**Conclusions:**

Patients with GLM exhibit disturbances in gut butanoic acid metabolism and significant differences in gut microbiota structure compared to healthy individuals.

## 1 Introduction

Granulomatous lobular mastitis (GLM) is a chronic inflammatory disease of the breast with an unknown etiology, classified as a type of non-puerperal mastitis (NPM). GLM is known for its prolonged course and tendency to recur, often requiring multiple surgeries. The most common clinical symptom is palpable breast lumps, often accompanied by pain (Martinez-Ramos et al., 2019). As the inflammation progresses, the lump may become painful and turn reddish in color, with the potential to cause abscess formation or the development of a sinus tract (Baslaim et al., 2007). Due to the unclear etiology of GLM, there are no specific targets for its management or prevention. Infection, inflammation, and hormonal disorders are considered triggers of the GLM (Sheybani et al., 2015). Clinical treatment options for GLM include glucocorticoids, antibiotics, methotrexate, prolactin-lowering agents, abscess drainage, and lesion resection (Baslaim et al., 2007; Yau et al., 2010; Lei et al., 2017). Among these, corticosteroids, surgery, and a combination of both are the most frequently used interventions (Zhang et al., 2019).

Currently, GLM is commonly considered an autoimmune disease, with varying degrees of immune dysfunction present during its course (Sheybani et al., 2015; Kong et al., 2020). The occurrence of extraintestinal autoimmune diseases was considered to be related to changes in the structure and function of gut microbiota, such as autoimmune arthritis, autoimmune encephalomyelitis, and type 1 diabetes (Kamada et al., 2013). The gut microbiota is considered an “external organ” involved in regulating immunity, which supports the development of the host immune system and promotes human metabolism and conversion of energy and nutrients (Vrieze et al., 2010). Researchers have transferred the gut microbiota from cows with mastitis to germ-free mice through fecal microbiota transplantation, creating a similar gut microbiota structure and inducing mastitis in the mouse (Ma et al., 2018). Gut microbiota and its metabolites can jointly regulate host immunity, maintain the integrity of the intestinal mucosal barrier, and promote intestinal homeostasis (Levy et al., 2017).

In our previous studies, we found significant differences in the gut microbiota characteristics between patients with NPM and healthy individuals using high-throughput sequencing. These differences were related to the production of short-chain fatty acid (SCFA), a type of intestinal metabolite (Dai et al., 2023). Therefore, this study aimed to further investigate the gut metabolic characteristics of SCFA in patients with GLM, a type of NPM. We collected fecal samples from patients with GLM to analyze the relationship between the content of SCFA in feces, gut microbiota composition, and other clinical indicators.

## 2 Methods

### 2.1 Collection of fecal specimens

Fecal specimens were collected from prospectively enrolled patients diagnosed with GLM and recruited healthy volunteers. All participants must have not used antibiotics, probiotics, glucocorticoids, immunosuppressants within the previous three months, have no acute or chronic gastrointestinal diseases, and sign an informed consent form. Before defecation, participants were instructed to empty their bladder to avoid urine contaminating the feces sample. A sterile container was placed in the toilet for the participants to defecate into. Subsequently, a sterile spoon was used to scoop out two full spoon portions (3–5 g) of the feces that had not come into contact with air. The sampled feces were placed into a sampling tube, and the lid was tightly sealed and stored in -80°C for testing.

### 2.2 Preparation of short-chain fatty acid standard solution

#### 2.2.1 Preparation of mixed standard

A total of 9,840 μl of n-butanol (HPLC grade) was added to a 15 ml centrifuge tube, followed by the sequential addition of an appropriate amount of eight different SCFA standards. The mixture was vortexed thoroughly to ensure even mixing, resulting in SCFA mixed standard stock solution A.

#### 2.2.2 Preparation of internal standard

A total of 9,990 μl of n-butanol (HPLC grade) was added into a 15 ml centrifuge tube, followed by the addition of 10 μl of the internal standard, 2-ethylbutyric acid. The solution was vortexed thoroughly to create internal standard stock solution.

### 2.3 Sample processing

A 20 mg fecal sample were added into a 2 ml grinding tube, and 800 μl of 0.5% phosphoric acid water (containing internal standard 2-ethylbutyric acid 10 μg/mL) was added. Subsequently, the samples were frozen ground for 3 min (50 HZ), then sonicated for 10 min, and then centrifugated at 4°C and 13,000 g for 15 min. Following this, 200 μl of the supernatant was transferred to 1.5 mL centrifuge tubes, and 200 μl of butanol was added for solvent extraction. The solution was vortexed for 10 s, subjected to low-temperature ultrasound for 10 min, centrifugated at 13,000 g at 4°C for 5 min, and transferred to an injection vial.

### 2.4 Gas chromatography-mass spectrometry detection

The analytical instrument used in this study was an 8890B-5977B GC/MSD gas chromatography-mass spectrometer (Agilent Technologies, Inc.). The chromatographic conditions were as follows: an HP FFAP capillary column (30 m × 0.25 mm × 0.25 μm. Agilent J&W Scientific, Folsom, CA, USA), with high-purity helium as the carrier gas (purity not less than 99.999%). The flow rate was set to 1.0 ml/min, and the temperature at the injection port was maintained at 180°C. The injection volume was 1 μl, using a split injection, with a split ratio of 10:1 and a solvent delay of 2.5 min. The programmed temperature gradient was as follows: the initial temperature of the column was set to 80°C, which increased to 120°C at a rate of 20°C/min, then to 160°C at a rate of 5°C/min, and finally maintained at 220°C for 3 min. Mass spectrometry conditions included an electron bombardment ion source (EI), with the ion source temperature set to 230°C, the quadrupole temperature at 150°C, and the transmission line temperature at 230°C. The electron energy was maintained at 70 eV, and a scanning method was used to select the ion-scanning mode (SIM).

Default parameters of the Mashunter quantitative software (Agilent, USA, version number: v10.0.707.0) were used to automatically identify and integrate the ionic fragments of the target SCFA, supplemented by manual inspection. The detection concentration of each sample was calculated using the standard curve, and the actual content of SCFA in the sample was determined.

### 2.5 High throughput sequencing analysis of gut microbiota

The E.Z.N.A.^®^ oil DNA kit (Omega Bio-tek, Norcross, GA, U.S.) were used to extract the total DNA of the microbial community. A 1% agarose gel electrophoresis was used to assess the quality of DNA extraction, while the concentration and purity of the DNA extraction were determine using a NanoDrop2000 spectrophotometer (Thermo Scientific Inc., USA). Primers 338F (5 ‘- ACTCCTACGGGGGGCAG-3’) and 806R (5 ‘- GACTACHVGGGTWTCTAAT-3’) were used for PCR amplification of the V3-V4 variable region of the 16S rRNA gene. The amplification procedure was as follows: pre-denaturation at 95°C for 3 min, followed by 27 cycles (denaturation at 95°C for 30 s, annealing at 55°C for 30 s, and extension at 72°C for 45 s), followed by stable extension at 72°C 10 min, and finally stored at 10°C (PCR instrument: ABI GeneAmp ^®^ 9700 type).

A 2% agarose gel was used to recover the PCR products, and the AxyPrep DNA Gel Extraction Kit (Axygen Biosciences, Union City, CA, USA) was used to purify the recovered products. A 2% agarose gel was used for electrophoretic detection, and the Quantus™ Fluorometer (Promega, USA) was used to detect and quantify the recovered products. The NEXTFLEX Rapid DNA-Seq Kit was employed to build a library. A MiSeq PE300 platform (Illumina) was used for sequencing (Shanghai Meiji Biomedical Technology Co., Ltd.).

The software fastp (Chen et al., 2018) and FLASH (Magoč and Salzberg, 2011) were used for quality control and splicing of the original sequencing data: (1) The base with tail mass value of less than 20 for reads and the reads with a quality control value of less than 50 bp were filtered; (2) Paired reads were merged with a minimum overlay length of 10 bp and a mismatch rate of no more than 0.2; (3) The samples were distinguished based on the barcodes and primers at the beginning and end of the sequence, and the sequence direction was adjusted with the barcode mismatch number of 0, and the maximum primer mismatch number of 2. Based on 97% similarity, UPARSE software was used to cluster operational taxonomic units (OTUs) and remove chimeras (Edgar, 2013; Stackebrandt and Goebel, 1994). The RDP classifier was used to annotate the species for each sequence (Wang et al., 2007). Compared to the Silva 16S rRNA database (v138), with a comparison threshold of 70%.

## 3 Results

A total of 61 participants were enrolled, including 35 in the GLM group and 26 in the HC group. Notably, no significant differences in age [(31.23 ± 4.62)years vs. (29.50 ± 5.62)years], height [(1.61 ± 0.05)m vs. (1.63 ± 0.05)m], weight [(60.91 ± 9.17)kg vs (59.38 ± 9.58)kg] and BMI (23.58 ± 3.28 vs. 22.36 ± 2.87) were observed between the two groups (p>0.05), indicating that they were comparable.

### 3.1 Characteristics of intestinal SCFA metabolism in the GLM group and HC group

**Figure 1:**
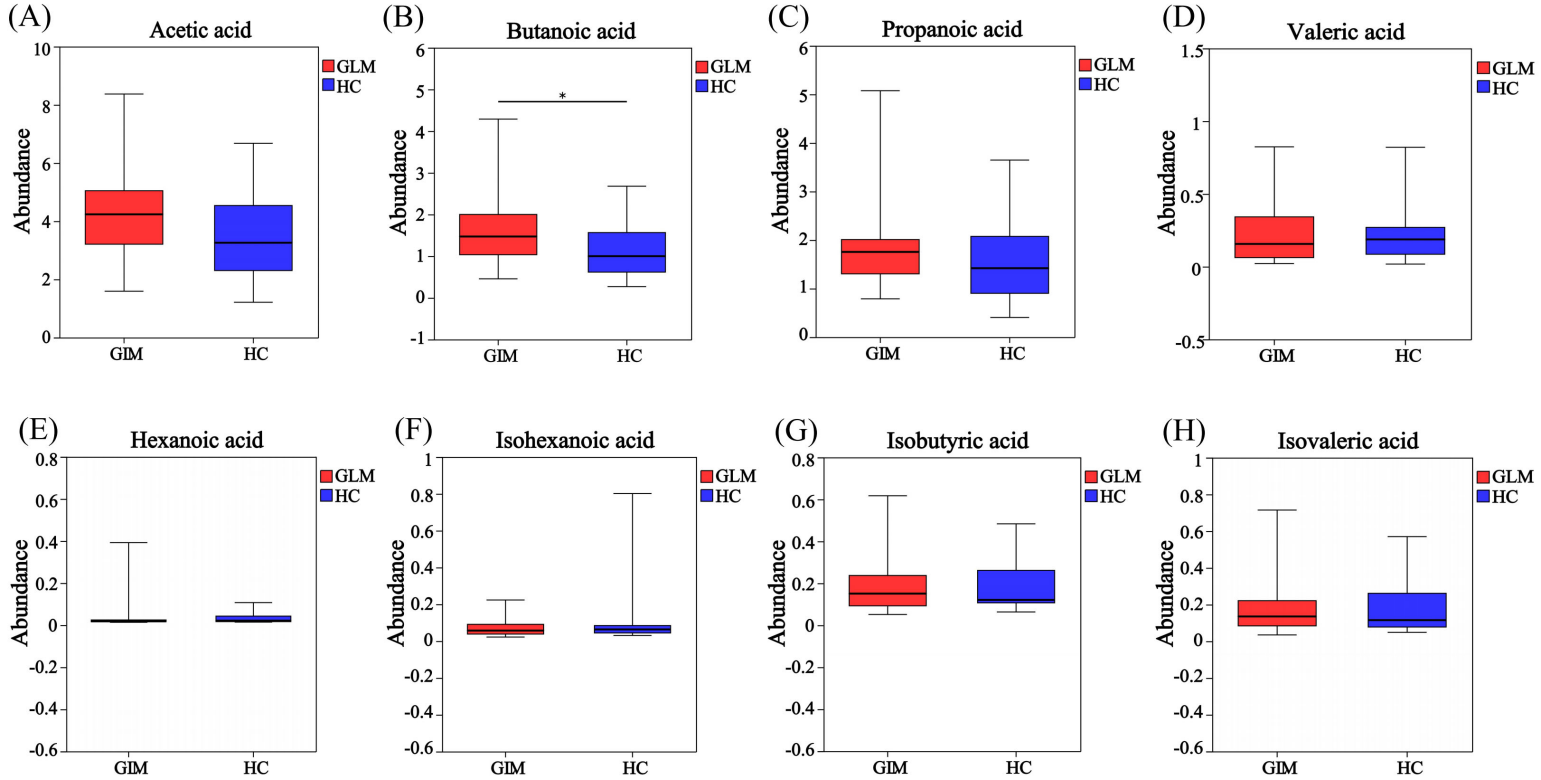
Box plot of SCFA difference analysis between GLM group and HC group. The levels of acetic acid **(A)**, butanoic acid **(B)**, propanoic acid **(C)**, valeric acid **(D)**, hexanoic acid **(E)**, isobutyric acid **(G)** and isovaleric acid **(H)** in the GLM group were higher than those in the HC group, while the level of isohexanoic acid **(F)** was lower in the GLM group. Red and blue represented the GLM (n=35) and HC subjects (n=26), respectively. *0.01<P<0.05.

GC-MS was used to measure specific SCFAs in the GLM and HC groups. The levels of acetic acid, propanoic acid, isobutyric acid, butanoic acid, isovaleric acid, valeric acid, and hexanoic acid levels were higher in the GLM group than in the HC group. There was a significant difference in butanoic acid content between the two groups[(1.59 ± 0.81)μg/mg vs (1.17 ± 0.67)μg/mg, *t*=2.23, *p*=0.03)] (**Table 1**, **Figure 1**).

**Table 1:** Content of SCFA in GLM group and HC group.

SCFA	Content(μg/mg)		T value	P value
	GLM	HC		
Acetic acid	4.20 ± 1.49	3.53 ± 1.57	1.67	0.10
Butanoic acid	1.59 ± 0.81	1.17 ± 0.67	2.23	0.03
Propanoic acid	1.85 ± 0.92	1.56 ± 0.81	1.33	0.19
Valeric acid	0.23 ± 0.21	0.20 ± 0.16	0.68	0.50
Hexanoic acid	0.05 ± 0.08	0.03 ± 0.03	0.68	0.50
Isohexanoic acid	0.07 ± 0.05	0.10 ± 0.15	-0.86	0.40
Isobutyric acid	0.19 ± 0.13	0.19 ± 0.12	0.19	0.85
Isovaleric acid	0.18 ± 0.15	0.17 ± 0.13	0.13	0.90

**Figure 2:**
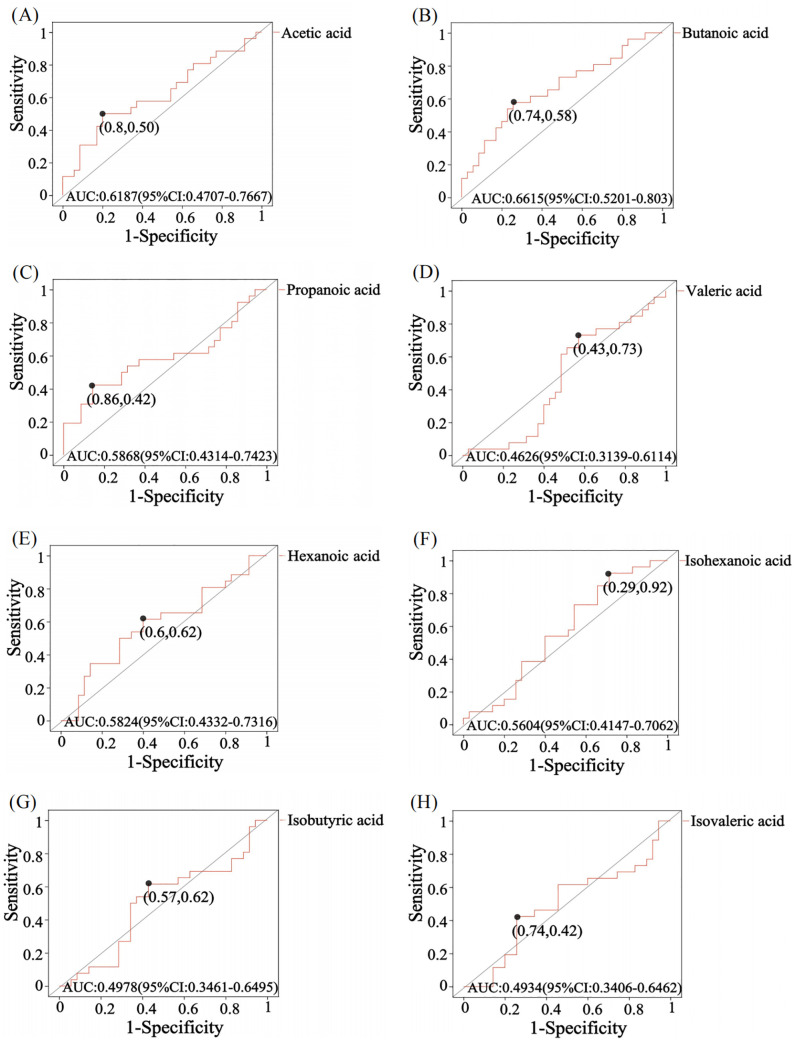
ROC analysis of SCFA in comparison between GLM and HC groups. The area under the curve (AUC) values for acetic acid **(A)**, butanoic acid **(B)**, propanoic acid **(C)**, hexanoic acid **(E)** and isohexanoic acid **(F)** were greater than 0.5, while the AUC values for valeric acid **(D)**, isobutyric acid **(G)** and isovaleric acid **(H)** were less than 0.5. The AUC value is usually between 0.5-1. In the case of AUC>0.5, the closer the AUC is to 1, the better the predictive effect on diseases.

**Figure 3:**
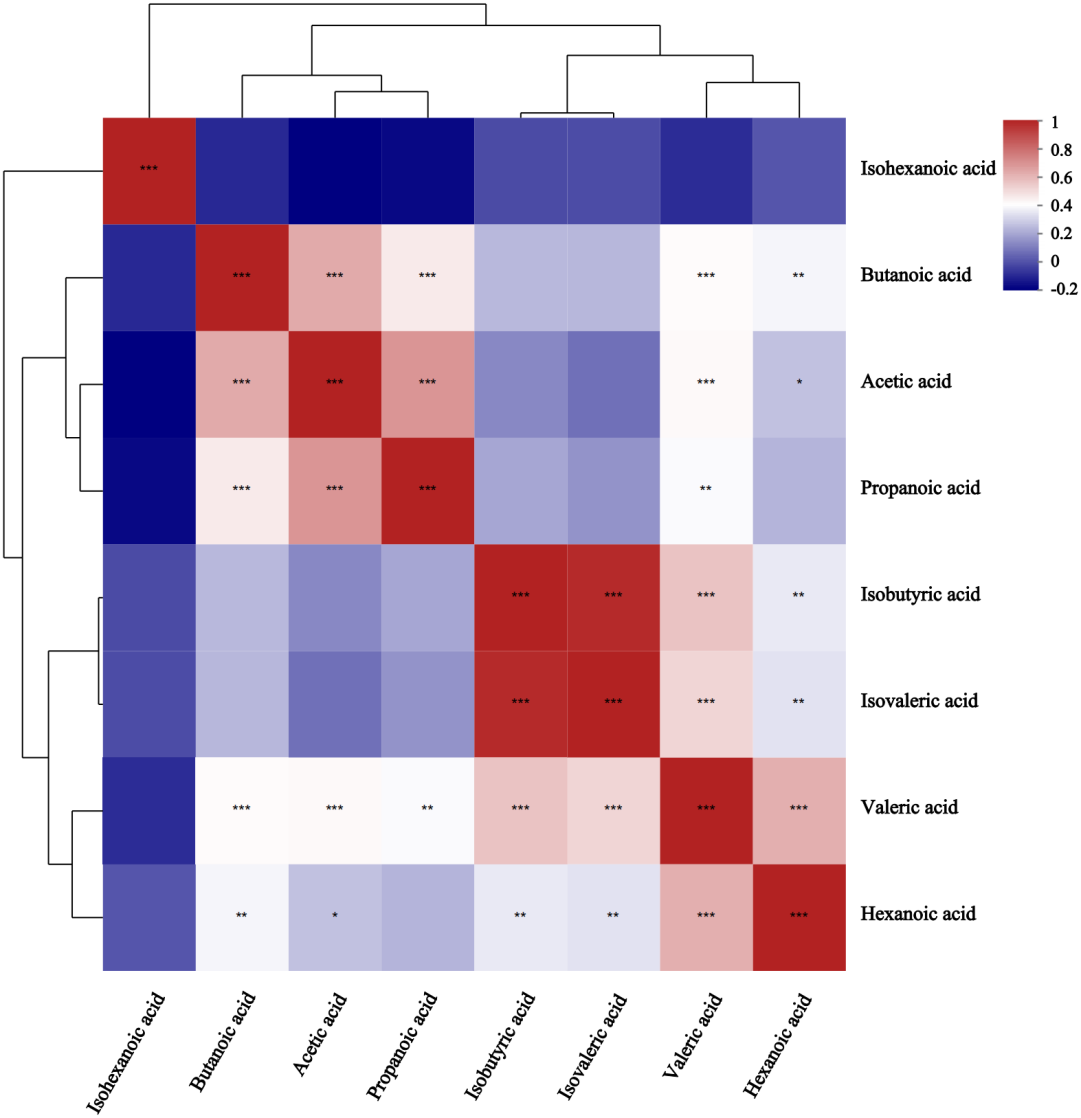
Correlation heatmap between metabolites in comparison between GLM and HC groups. Different colors represent the magnitude of the correlation coefficient, and the closer the absolute value is to 1, the higher the positive or negative correlation of metabolites.* Represents P-value<0.05,indicating significant correlation; ** represents P-value<0.01; *** represents P-value<0.001, indicating extremely significant correlation.

Receiver operating characteristic (ROC) analysis was used to further validate key metabolites that significant impacted differentiation between the two groups. The larger the area under the curve (AUC), the better the predictive effect for diseases. The AUC value for butanoic acid was greater than 0.5(AUC=0.66,95%CI:0.52-0.80) (**Figure 2**), suggesting that it could be considered a potential biomarker with diagnostic significance for the disease. Metabolite correlation analysis showed that the potential biomarker, butanoic acid, was positively correlated with acetic acid, propanoic acid, valeric acid, and hexanoic acid in feces samples (**Figure 3**).

**Figure 4:**
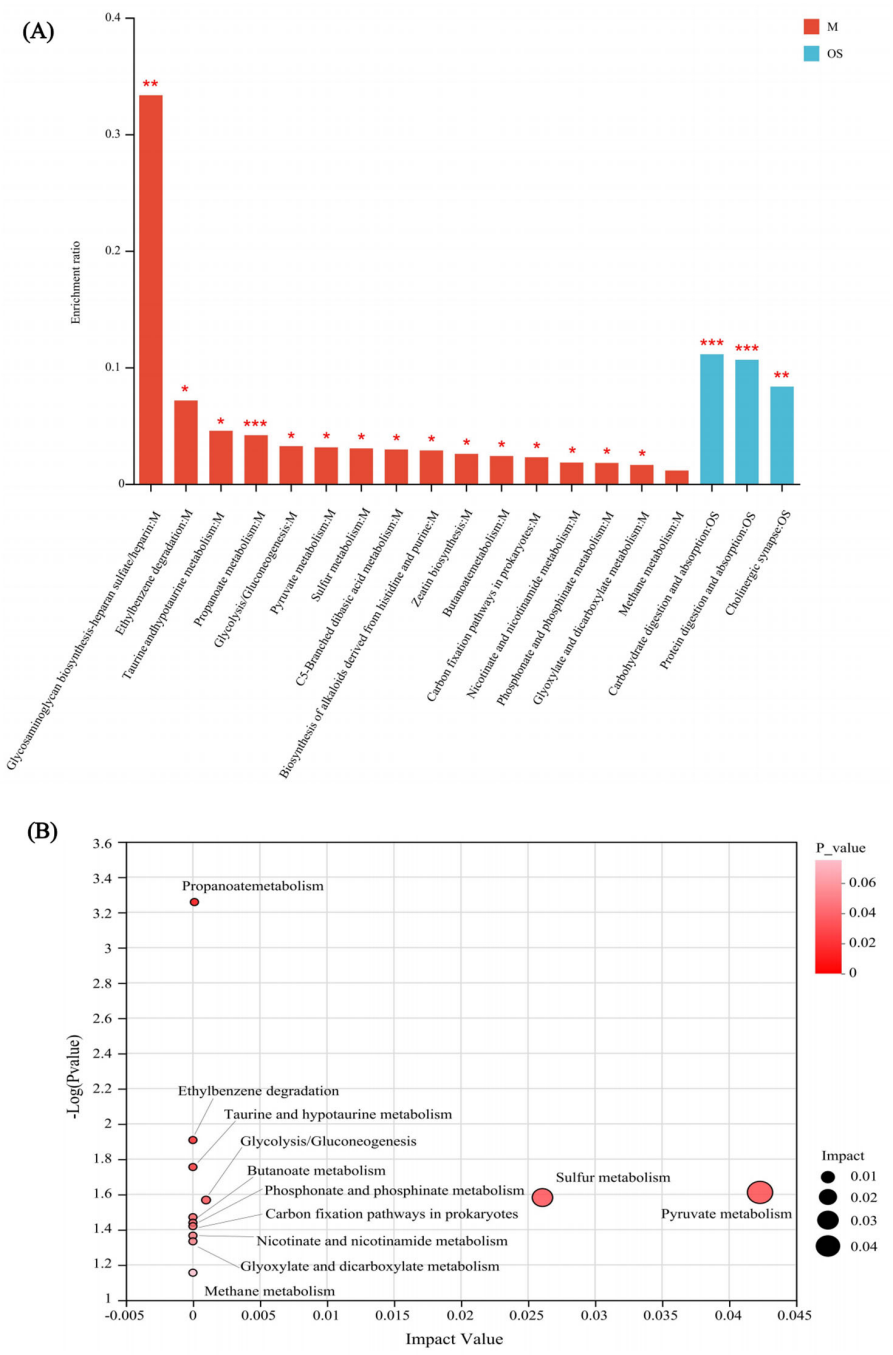
KEGG enrichment analysis **(A)** and topology analysis **(B)** of SCFA in comparison between GLM and HC groups M, Metabolism; OS, Organismal Systems. *0.01<P<0.05, **0.001 < P ≤ 0.01, ***P≤0.001.

Enrichment analysis revealed significant differences in metabolic pathways between the HC and GLM groups, including carbohydrate digestion and absorption, protein digestion and absorption, cholinergic synapse pathways in organismal systems, propanoate metabolism, glycosaminoglycan biosynthesis-heparansulfate/heparin, ethylbenzene degradation, taurine and hypotaurine metabolism, glycolysis/Gluconeogenesis, pyruvatemetabolism, sulfur metabolism, C5-Branched dibasic acid metabolism, biosynthesis of alkaloids derived from histidine and purine, zeatin biosynthesis, butanoate metabolism, carbon fixation pathways in prokaryotes, nicotinate and nicotinamide metabolism, phosphonate and phosphinate metabolism, glyoxylate and dicarboxylate metabolism in metabolism category, had significant differences between the HC and GLM groups (**Figure 4**).

**Figure 5:**
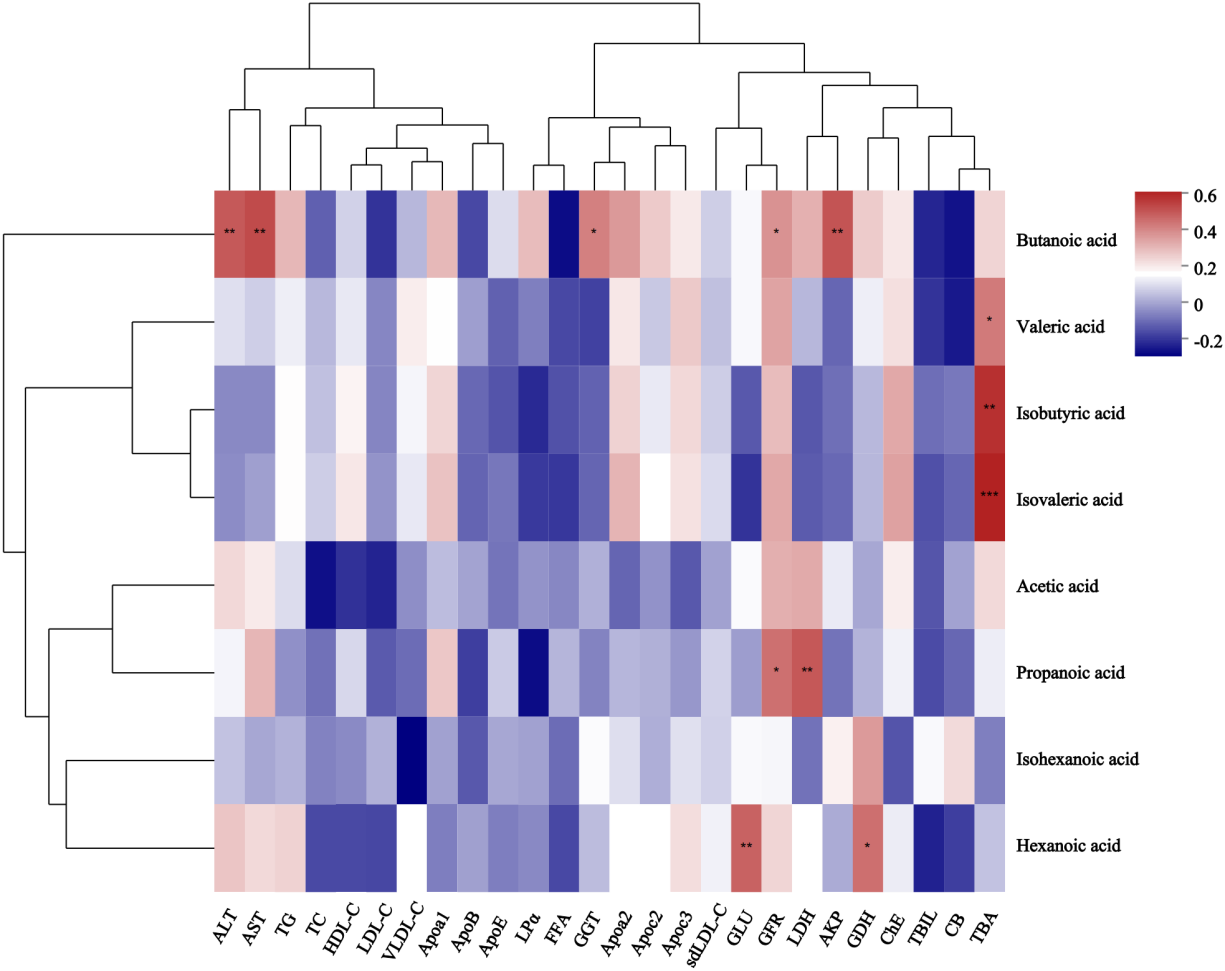
Correlation heatmap between SCFAs and related clinical features of GLM. *0.01<P<0.05, **0.001 < P ≤ 0.01, ***P≤0.001. After verification, it was found that the correlation P-value between isovaleric acid and TBA in the figure is also less than or equal to 0.001.

Correlation heatmap analysis was used to calculate the correlation coefficient between SCFAs and relevant clinical factors, and the results were displayed as a heatmap (**Figure 5**). These clinically relevant factors included alanine aminotransferase (ALT), aspartate aminotransferase (AST), triglyceride (TG), total cholesterol (TC), high-density lipoprotein cholesterol (HDL-C), low density lipoprotein cholesterol (LDL-C), very low-density lipoprotein cholesterol (VLDL-C), apolipoprotein A1 (Apoa1), apolipoprotein B (ApoB), apolipoprotein E (ApoE), Lipoprotein alpha (LPα), free fatty acids (FFA), gamma-glutamyl transpeptidase(GGT), apolipoprotein A2 (Apoa2), apolipoprotein C2(Apoc2), apolipoprotein C3 (Apoc3), small and dense lipoprotein cholesterol (sdLDL-C), glucose (GLU), glomerular filtration rate (GFR), lactic dehydrogenase (LDH), alkaline phosphatase (AKP), glutamic dehydrogenase (GDH), Cholinesterase (ChE), total bilirubin (TBIL), conjugated bilirubin (CB), total bile acid (TBA). The butanoic acid content in the feces was positively correlated with the levels of ALT, AST, GGT, GFR, and AKP in the peripheral blood of patients with GLM. Hexanoic acid content was positively correlated with GLU and GDH levels. Propanoic acid content was positively correlated with GFR and LDH levels. Valeric acid, isobutyric acid, and isovaleric acid contents were positively correlated with TBA levels.

### 3.2 Characteristics of gut microbiota in GLM and their relationship with metabolism of SCFA

#### 3.2.1 Sequencing data information

A total of 3059051 valid sequences were measured, and the length distribution of the trimmed sequences ranged from 410 to 440. Based on the minimum number of sample sequences, 899 OTUs were identified with 97% sequence similarity. OTU taxonomic analysis revealed that the feces samples from the two groups contained a total of 579 species.

#### 3.2.2 Species difference and SCFA correlation analysis

The alpha diversity analysis showed that the Chao(301.29 ± 73.66 vs 265.63 ± 59.81), Sobs(252 ± 64.74 vs 215.03 ± 48.47), Shannon(3.40 ± 0.42 vs 3.09 ± 0.39), Shannoneven(0.62 ± 0.06 vs 0.58 ± 0.07), and Invsimpson(16.12 ± 7.89 vs 11.93 ± 5.18) indices in the HC group were significantly higher than those in the GLM group(p<0.05), and the ACE(298.05 ± 72.40 vs 268.6 ± 61.44) and coverage(1.00 ± 0.0004 vs 1.00 ± 0.0004) indices did not differ significantly between the two groups (p>0.05) (**Table 2**).

**Table 2:** Alpha diversity analysis of gut microbiota in GLM group and HC group.

Diversity index	Group		T value	P value
	HC	GLM		
chao	301.29 ± 73.66	265.63 ± 59.81	2.09	<0.05
ace	298.05 ± 72.40	268.60 ± 61.44	1.72	>0.05
sobs	252 ± 64.74	215.03 ± 48.47	2.55	<0.05
shannon	3.40 ± 0.42	3.09 ± 0.39	3.01	<0.05
shannoneven	0.62 ± 0.06	0.58 ± 0.07	2.44	<0.05
invsimpson	16.12 ± 7.89	11.93 ± 5.18	2.50	<0.05
coverage	1.00 ± 0.0004	1.00 ± 0.0004	-0.04	>0.05

**Figure 6:**
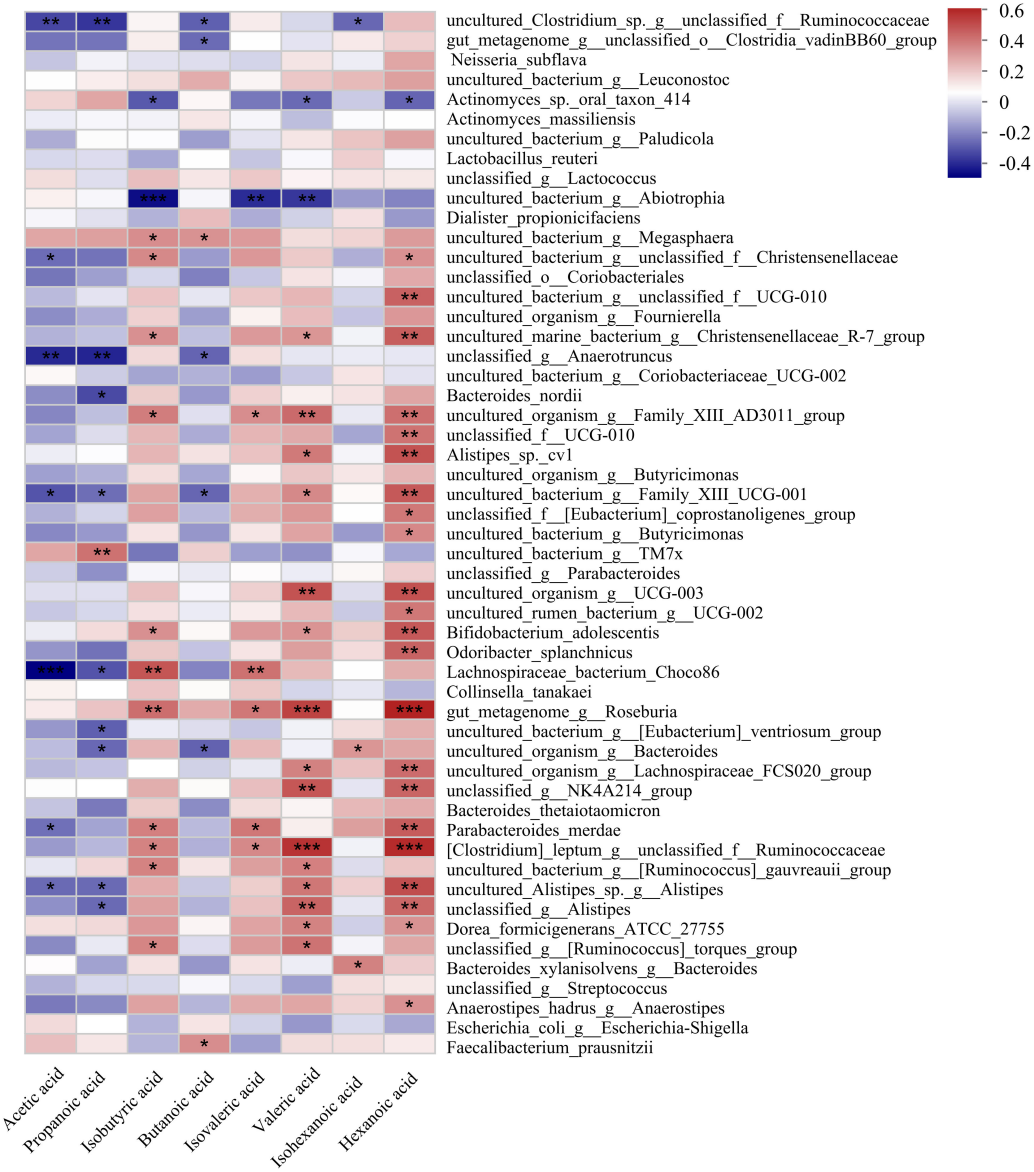
Correlation heatmap of gut microbiota and SCFA metabolites in GLM and HC group at species level. *0.01<P<0.05, **0.001 < P ≤ 0.01, ***P≤0.001.

Analysis of intergroup differences revealed significant differences in the abundance of 53 microorganisms between the two groups at the species level (**Supplementary Table 1**). Correlation heatmap analysis was used to determine the correlation between these 53 microorganisms and SCFAs levels, of which the results were presented using correlation heatmap (**Figure 6**). The levels of Faecalibacteriumprausnitzii and uncultured_bacterium_g:Megasphaera were positively correlated with butyrate in feces. Uncultured_Clostridium_sp._g:unclassified_f:Ruminococcaceae, gut_metagenome_g:unclassified_o:Clostridia_vadinBB60_group, unclassified_g:Anaerotruncus、uncultured_bacterium_g:Family_XIII_UCG-001, and uncultured_organism_g:Bacteroides were negatively correlated with butyrate levels.

### 3.3 Difference of SCFA in GLM patients with different prognosis

**Figure 7:**
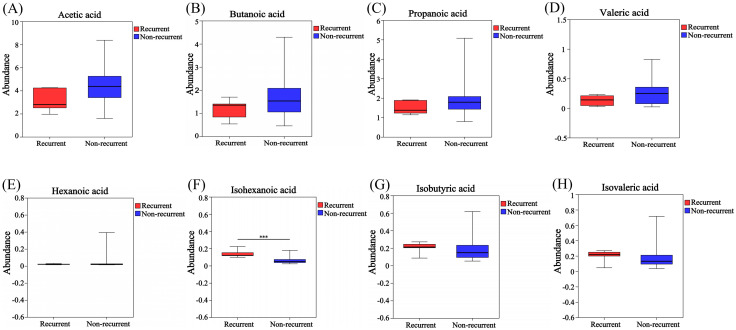
Analysis of differences in SCFA between recurrent and non-recurrent groups of GLM. The levels of acetic acid **(A)**, butanoic acid **(B)**, propanoic acid **(C)**, valeric acid **(D)** and hexanoic acid **(E)** in the recurrent group were lower than those in the non- recurrent group, while the levels of isohexanoic acid **(F)**, isobutyric acid **(G)** and isovaleric acid **(H)** were higher in the recurrent group.Red and blue represented the recurrent (n=5) and non-recurrent (n=29) GLM patients, respectively. ***P≤0.001.

**Figure 8:**
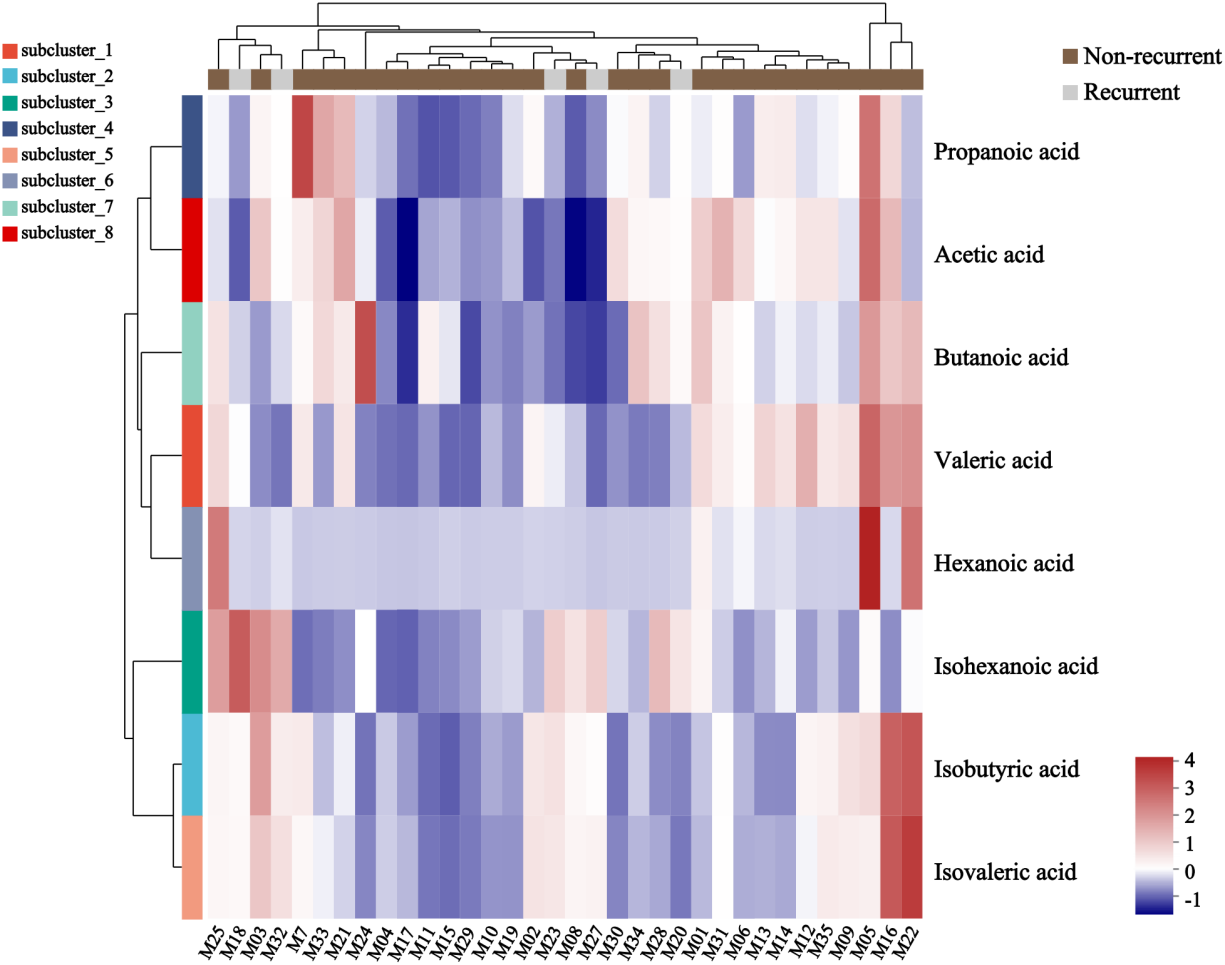
Heatmap of metabolite cluster analysis in recurrent and non-recurrent groups of GLM. Each column in the figure represented a sample, and each row represented a metabolite. The colors in the figure indicated the relative expression levels of metabolites in the group of samples. The specific trend of expression level changes can be seen in the numerical annotation under the color bar in the lower right corner.

A total of 34 GLM patients were followed-up for 1 year, of which five patients experienced recurrence. There were no significant differences in age[(31.80 ± 3.03)years vs (31.24 ± 4.91)years], height[(1.60 ± 0.06)m vs (1.61 ± 0.04)m], weight[(59.80 ± 7.98)kg vs (61.59 ± 9.24)kg] or BMI (23.34 ± 2.64 vs 23.77 ± 3.37) between the recurrent and non-recurrent group (p>0.05). Differential analysis showed that the isohexanoic acid levels in the recurrent group were significantly higher than those in the non-recurrent group (**Table 3**, **Figure 7**). A metabolic profile was created based on the results of the difference analysis between the two groups of SCFA, and cluster analysis was performed (**Figure 8**). ROC analysis (**Supplementary Figure 1**) showed the AUC value of isohexanoic acid was 0.9172(95%CI:0.8218-1), indicating well diagnostic potential.

**Table 3:** Content of SCFA in recurrent group and non-recurrent group of GLM.

SCFA	Content(μg/mg)		T value	P value
	Recurrent	Non-recurrent		
Acetic acid	3.15 ± 1.04	4.35 ± 1.52	-1.69	0.10
Butanoic acid	1.16 ± 0.47	1.69 ± 0.84	-1.33	0.19
Propanoic acid	1.50 ± 0.36	1.93 ± 0.99	-0.94	0.36
Valeric acid	0.13 ± 0.09	0.25 ± 0.22	-1.24	0.22
Hexanoic acid	0.02 ± 0.01	0.05 ± 0.09	-0.72	0.48
Isohexanoic acid	0.14 ± 0.05	0.06 ± 0.04	4.10	0.0003
Isobutyric acid	0.20 ± 0.07	0.19 ± 0.14	0.12	0.91
Isovaleric acid	0.20 ± 0.09	0.18 ± 0.16	0.25	0.80

## 4 Discussion

In this study, we collected fecal samples from diseased and healthy individuals for targeted metabolomic testing, which provided a partial insight into the metabolism of SCFAs *in vivo*. SCFAs in the large intestine are primarily produced by bacterial metabolism of complex carbohydrates and are also derived from the breakdown of proteins and peptides (Macfarlane and Macfarlane, 2003). Most SCFAs are absorbed in the colon and cecum, while 5%–10% are excreted from the body through feces (Wong et al., 2006; McNeil et al., 1978). SCFAs play a role in both the intestinal lumen and mucosa through colon cell metabolism, thereby promoting colon function and preventing disease (Topping and Clifton, 2001). In terms of function, SCFAs, as important metabolites of the gut microbiota, are related to host health. They regulate intestinal signal transduction, motility, and pH (Nicholson et al., 2012). In addition, SCFAs are involved in the metabolism of carbohydrate and lipid (Ríos-Covián et al., 2016). Moreover, they regulate the expression of inflammatory cytokines in intestinal epithelial cells by activating the MEK-ERK and p38 MAPK signaling pathways, which promotes the function of GPR41 and GPR43 receptors on the surface of colon epithelial cells, helping to maintain gut homeostasis (Campos-Perez and Martinez-Lopez, 2021). SCFAs also stimulate colonic peristalsis, by promoting the release of serotonin 5-HT and neuronal CGRP (Grider and Piland, 2007). The main SCFA in the colon includes acetate, propionate, and butyrate in a molar ratio of approximately 60:20:20 (Levy et al., 2017; Grider and Piland, 2007).

The results of this study showed that the butanoic acid content in the feces of patients with GLM was significantly higher than that in healthy individuals. The levels of acetic acid, propanoic acid, isobutyric acid, isovaleric acid, valeric acid, and hexanoic acid were also higher in the GLM group than in the HC group; however, the differences were not statistically significant. KEGG pathway enrichment analysis showed digestion and absorption of carbohydrates and proteins as well as some metabolic pathways in the gut of patients with GLM. Butyrate-producing bacteria can produce butyrate through the butyrate kinase and butyryl-CoA pathways, which are mainly metabolized by the colon epithelial cells after production, whereas other SCFAs are mostly metabolized by the liver (Ríos-Covián et al., 2016; Koh et al., 2016). In addition to supplying energy to intestinal epithelial cells, and maintaining the integrity of the intestinal mucosal barrier and intestinal homeostasis (Lv et al., 2022), butyric acid regulates the secretion of certain hormones, cell differentiation and proliferation, and inflammation/immune processes (Vinolo et al., 2011). Both butyrate and propionate have been shown to inhibit diet-induced obesity, promote intestinal hormone production, and reduce food intake (Lin et al., 2012). Chronic inflammation is believed to be related to the bioavailability of butyrate (Vrieze et al., 2010). Clinical studies have shown that excessive levels of butyrate in feces and colonic dialysate are associated with the occurrence of neonatal necrotizing colitis and the severity of ulcerative colitis, respectively (Szylit et al., 1998; Roediger et al., 1982). Similarly, the absorption function of the intestinal mucosa in patients with non-specific inflammatory bowel disease was also found to be impaired (Hawker et al., 1980). In the intestinal mucosa of patients with non-specific inflammatory bowel disease, the rate of butyric acid oxidation was significantly reduced (Chapman et al., 1994, Chapman et al., 1995), which is related to the reduced expression of the butyric acid transporter MCT1 in the intestinal mucosa (Thibault et al., 2010). Similar situations have also been observed in the feces of patients with obesity, in which the SCFA contents were higher than those in healthy individuals, and the fecal SCFA contents in healthy individuals were inversely proportional to their intestinal absorption rate; this suggests that the relatively high amount of SCFAs in the feces of patients with obesity may be due to reduced intestinal absorption; however, further evidence is needed to confirm this hypothesis (Rahat-Rozenbloom et al., 2014). *In vitro* studies have shown that SCFA exhibits neutrophil chemotaxis and inhibits the chemotaxis of other inducible factors in neutrophils (Vinolo et al., 2011). In addition, butyrate was found to inhibit LPS-induced macrophage migration (Maa et al., 2010). Therefore, the increase in butanoic acid content observed in patients with GLM in this study may be due to abnormal colon utilization of butyrate, and further experiments are needed to confirm this hypothesis.

In this study, the levels of butanoic acid in the feces of patients with GLM were positively correlated with peripheral blood ALT, AST, GGT, GFR-EPI, and AKP levels, whereas the levels of hexanoic acid and propanoic acid were correlated with GLU and GFR-EPI, respectively. This association may be related to the metabolic pathways of SCFAs. SCFAs are metabolized by cells of the colon and liver after absorption, whereas unmetabolized SCFAs are excreted from the body (Lin et al., 2012). Gut-derived SCFAs are substrates for gluconeogenesis and palmitate and cholesterol synthesis in the liver (den Besten et al., 2013). Bloemen et al. (2009) reported that most of the propanoic acid and butyric acid released from the intestine is metabolized by the liver, while acetic acid is absorbed by peripheral tissues, such as adipose tissue and muscle. Lv et al. (2021) found that Lactobacillus acidophilus can reduce the elevation of serum ALT, aspartate aminotransferase, alkaline phosphatase, and bile acid induced by D-galactosamine in rats, while also correcting the metabolic disorder of butyric acid in the intestine. Various SCFA interventions have been reported to reduce fasting blood glucose, total cholesterol, and triglyceride levels in diabetic mice (Zheng et al., 2024). The production of SCFAs is influenced by the microbial community in the intestine, substrates, and time of intestinal transport (Wong et al., 2006). Furthermore, disturbances in the gut microbiota and serum metabolism are associated with liver and intestinal damage (Zhuang et al., 2023).

Moreover, characteristics of gut microbiota were also analyzed. The alpha diversity analysis showed that the index of Chao, Sobs, Shannon, Shannoneven and Invsimpson in healthy individuals were higher than those in GLM patients, which indicated the richness, evenness, and diversity of gut microbiota in healthy individuals are higher than those in GLM patients. These results were basically consistent with our previous study on the characteristics of gut microbiota in patients with NPM (Dai et al., 2023).This study focused on the differences in composition of gut microbiota at the species level, and explored the correlation between these differential species and SCFAs. Among them, Faecalibacterium_prausnitzii and uncultured_bacterium_g:Megasphaera were positively correlated with butanoic acid in feces, whereas uncultured_Clostridium_sp._g:unclassified_f:Ruminococcaceae, gut_metagenome_g:unclassified_o:Clostridia_vadinBB60_group, unclassified_g:Anaerotruncus, uncultured_bacterium_g:Family_XIII_UCG-001, and uncultured_organism_g:Bacteroides were negatively correlated with butanoic acid. Faecalibacteriumprausnitzii, which belongs to the Clostridium cluster IV, is a butyrate-producing bacterium that can colonize the mucus layer and improve colonic butyrate utilization (Karim et al., 2024; Oliphant and Allen-Vercoe, 2019). Megasphaera belongs to the phylum Firmicutes, and Vania et al. reported that patients with obesity had improved obesity-related indicators following biliointestinal surgery, while the levels of Megasphaera in the feces increased and the levels of Clostridiaceae and Ruminococcaceae decreased (Patrone et al., 2016). Anaerotruncus belongs to the Ruminococcaceae family and phylum Firmicutes and is a butyrate-producing bacterium (Louis and Flint, 2009). Family_ XIII_ UCG-001 belongs to the Anaerovoracaceae family and Firmicutes phylum. Limited research has shown that Family_ XIII_ UCG-001 in the gut microbiota has a decreased abundance in rats with relieved rheumatoid arthritis and in patients with classical homocystinuria (Li et al., 2022; Rizowy et al., 2020). In another animal experiment, alcoholic liver-like changes were alleviated in mice treated with ganoderic acid A, along with increased levels of butyric acid and Family_XIII_UCG_001 in the intestine (Lv et al., 2022). We also analyzed the different prognostic perspectives and found that fecal samples from patients with recurrence had higher levels of isohexanoic acid; however, this comparison involves five patients with recurrence and 29 patients without recurrence, resulting in a significant difference in sample size; therefore, these results should be interpreted with caution. Irving et al. (2016) reported that an increase in serum isohexanoic acid levels is associated with higher BMI.

However, this study had certain limitations. Our data reflected the contents of SCFAs in the feces of patients with GLM and revealed some correlations with clinical characteristics; nonetheless, it did not capture the relationship between SCFAs and intestinal permeability owing to the limitations of clinical practice. Therefore, further experiments are required to confirm whether the observed increase in butanoic acid content in patients with GLM is due to abnormal utilization of butyrate by the colon or excessive production.

In conclusion, our findings revealed that patients with GLM exhibited disrupted intestinal butyrate metabolism and significant differences in intestinal microbiota structure. GC-MS analysis showed that the feces of patients with GLM had significantly higher contents of SCFA butanoic acid compared to healthy individuals, suggesting that this metabolite play a significant role in differentiating the two groups. Furthermore, high-throughput sequencing results showed significant differences in the gut microbiota between patients with GLM and healthy individuals; notably, patients with GLM had significantly lower abundance and diversity of gut microbiota compared to healthy individuals, and a total of 53 bacterial species showing significant differences in abundance. This study provides insight into the pathological characteristics of patients with GLM from the unique perspective of gut metabolism and microbiota. However, further research is needed to explore the relationship between gut microenvironment and disease prognosis.

## Data Availability

The sequencing analysis data generated in this study have been deposited in https://www.jianguoyun.com/p/DV_UT5MQr9C7DRii0fkFIAA. All other relevant data are within the manuscript and in **Supplementary Material**.
