# Supplementary material for: Characteristics of metabolites analysis for patients with granulomatous lobular mastitis
Source: Front Cell Infect Microbiol. 2025 Jun 10;15:1514315. doi: 10.3389/fcimb.2025.1514315 (PMC12186243; doi:10.3389/fcimb.2025.1514315)
Supplement: Supplementary file 1 [file Table1.docx]

Supplementary Material

**Supplementary Table 1.** Mean relative abundance of microorganisms at species level differences in gut microbiota structure between GLM and HC groups

| Species name | Mean relative abundance(%) | | Z value | P value |
| --- | --- | --- | --- | --- |
|  | GLM | HC |  |  |
| s__Faecalibacterium_prausnitzii | 12.36±9.52 | 8.29±7.43 | 4.07 | ＜0.05 |
| s__Escherichia_coli_g__Escherichia-Shigella | 4.61±9.52 | 1.13±2.92 | 3.48 | ＜0.05 |
| s__Anaerostipes_hadrus_g__Anaerostipes | 1.32±2.11 | 4.24±6.60 | -2.92 | ＜0.05 |
| s__unclassified_g__Streptococcus | 0.78±0.91 | 2.47±4.91 | -1.68 | ＜0.05 |
| s__Bacteroides_xylanisolvens_g__Bacteroides | 0.36±0.70 | 0.94±1.40 | -0.58 | ＜0.01 |
| s__unclassified_g__[Ruminococcus]_torques_group | 0.21±0.42 | 0.69±0.82 | -0.48 | ＜0.01 |
| s__Dorea_formicigenerans_ATCC_27755 | 0.32±0.50 | 0.55±0.62 | -0.23 | ＜0.05 |
| s__uncultured_Alistipes_sp._g__Alistipes | 0.21±0.65 | 0.52±0.65 | -0.31 | ＜0.01 |
| s__unclassified_g__Alistipes | 0.32±0.94 | 0.37±0.54 | -0.05 | ＜0.05 |
| s__uncultured_bacterium_g__[Ruminococcus]_gauvreauii_group | 0.18±0.24 | 0.38±0.40 | -0.20 | ＜0.05 |
| s__Parabacteroides_merdae | 0.11±0.16 | 0.35±0.40 | -0.24 | ＜0.01 |
| s__Bacteroides_thetaiotaomicron | 0.10±0.23 | 0.29±0.41 | -0.20 | ＜0.01 |
| s__[Clostridium]_leptum_g__unclassified_f__Ruminococcaceae | 0.03±0.10 | 0.32±0.84 | -0.29 | ＜0.01 |
| s__unclassified_g__NK4A214_group | 0.06±0.17 | 0.19±0.32 | -0.13 | ＜0.05 |
| s__uncultured_organism_g__Bacteroides | 0.06±0.23 | 0.17±0.41 | -0.10 | ＜0.05 |
| s__uncultured_organism_g__Lachnospiraceae_FCS020_group | 0.10±0.21 | 0.11±0.12 | -0.01 | ＜0.05 |
| s__gut_metagenome_g__Roseburia | 0.02±0.05 | 0.14±0.38 | -0.13 | ＜0.05 |
| s__uncultured_bacterium_g__[Eubacterium]_ventriosum_group | 0.04±0.18 | 0.12±0.28 | -0.08 | ＜0.05 |
| s__Collinsella_tanakaei | 0 | 0.16±0.79 | -0.16 | ＜0.05 |
| s__Lachnospiraceae_bacterium_Choco86 | 0.03±0.04 | 0.08±0.12 | -0.06 | ＜0.01 |
| s__Bifidobacterium_adolescentis | 0.03±0.11 | 0.06±0.11 | -0.03 | ＜0.05 |
| s__Odoribacter_splanchnicus | 0.02±0.04 | 0.07±0.11 | -0.04 | ＜0.05 |
| s__unclassified_g__Parabacteroides | 0.0030±0.0056 | 0.07±0.18 | -0.07 | ＜0.01 |
| s__uncultured_rumen_bacterium_g__UCG-002 | 0.02±0.07 | 0.04±0.10 | -0.02 | ＜0.05 |
| s__uncultured_organism_g__UCG-003 | 0.02±0.03 | 0.05±0.06 | -0.03 | ＜0.05 |
| s__uncultured_bacterium_g__TM7x | 0.04±0.06 | 0.01±0.02 | 0.03 | ＜0.01 |
| s__uncultured_bacterium_g__Butyricimonas | 0.01±0.04 | 0.04±0.06 | -0.03 | ＜0.05 |
| s__unclassified_f__[Eubacterium]_coprostanoligenes_group | 0.02±0.14 | 0.02±0.06 | 0.0035 | ＜0.05 |
| s__uncultured_bacterium_g__Family_XIII_UCG-001 | 0.01±0.02 | 0.03±0.05 | -0.02 | ＜0.01 |
| s__uncultured_organism_g__Butyricimonas | 0.01±0.03 | 0.03±0.04 | -0.02 | ＜0.05 |
| s__Alistipes_sp._cv1 | 0.01±0.05 | 0.03±0.04 | -0.01 | ＜0.05 |
| s__unclassified_f__UCG-010 | 0.0026±0.0083 | 0.03±0.14 | -0.03 | ＜0.05 |
| s__uncultured_organism_g__Family_XIII_AD3011_group | 0.01±0.03 | 0.02±0.03 | -0.01 | ＜0.05 |
| s__Bacteroides_nordii | 0.01±0.03 | 0.02±0.03 | -0.01 | ＜0.05 |
| s__unclassified_g__Anaerotruncus | 0.01±0.01 | 0.02±0.02 | -0.01 | ＜0.05 |
| s__uncultured_bacterium_g__Coriobacteriaceae_UCG-002 | 0.02±0.11 | 0 | 0.02 | ＜0.05 |
| s__uncultured_marine_bacterium_g__Christensenellaceae_R-7_group | 0.0011±0.0022 | 0.02±0.04 | -0.02 | ＜0.05 |
| s__unclassified_o__Coriobacteriales | 0.0027±0.0115 | 0.01±0.02 | -0.01 | ＜0.01 |
| s__uncultured_bacterium_g__unclassified_f__UCG-010 | 0.01±0.04 | 0.0045±0.0126 | 0.0021 | ＜0.05 |
| s__uncultured_organism_g__Fournierella | 0.0014±0.0040 | 0.0086±0.0197 | -0.01 | ＜0.05 |
| s__uncultured_bacterium_g__Megasphaera | 0 | 0.01±0.02 | -0.01 | ＜0.05 |
| s__uncultured_bacterium_g__unclassified_f__Christensenellaceae | 0.0023±0.0065 | 0.01±0.01 | -0.0028 | ＜0.05 |
| s__Dialister_propionicifaciens | 0.01±0.02 | 0.0004±0.0015 | 0.0050 | ＜0.01 |
| s__uncultured_bacterium_g__Abiotrophia | 0.0034±0.0076 | 0.0010±0.0039 | 0.0023 | ＜0.05 |
| s__unclassified_g__Lactococcus | 0.0024±0.0097 | 0.0011±0.0017 | 0.0013 | ＜0.05 |
| s__Actinomyces_massiliensis | 0.0009±0.0020 | 0.0019±0.0025 | -0.0010 | ＜0.05 |
| s__Lactobacillus_reuteri | 0.0025±0.0059 | 0.0003±0.0010 | 0.0022 | ＜0.05 |
| s__uncultured_bacterium_g__Paludicola | 0.0005±0.0017 | 0.0023±0.0048 | -0.0018 | ＜0.05 |
| s__Actinomyces_sp._oral_taxon_414 | 0.0014±0.0020 | 0.0003±0.0009 | 0.0011 | ＜0.05 |
| s__uncultured_bacterium_g__Leuconostoc | 0 | 0.0014±0.0064 | -0.0014 | ＜0.05 |
| s__Neisseria_subflava | 0 | 0.0005±0.0017 | -0.0005 | ＜0.05 |
| s__uncultured_Clostridium_sp._g__unclassified_f__Ruminococcaceae | 0 | 0.0003±0.0008 | -0.0003 | ＜0.05 |
| s__gut_metagenome_g__unclassified_o__Clostridia_vadinBB60_group | 0 | 0.0002±0.0007 | -0.0002 | ＜0.05 |

**Supplementary Figure 1.** ROC analysis of SCFA in comparison between recurrent and non-recurrent groups of GLM
